# Effect of 905 MHz microwave radiation on colony growth of the yeast *Saccharomyces cerevisiae* strains FF18733, FF1481 and D7

**DOI:** 10.2478/v10019-010-0019-7

**Published:** 2010-05-24

**Authors:** Ivana Vrhovac, Reno Hrascan, Jasna Franekic

**Affiliations:** Faculty of Food Technology and Biotechnology, University of Zagreb, Zagreb, Croatia

**Keywords:** microwave radiation, *Saccharomyces cerevisiae*, colony growth

## Abstract

**Background:**

The aim of this study was to evaluate the effect of weak radiofrequency microwave (RF/MW) radiation emitted by mobile phones on colony growth of the yeast *Saccharomyces cerevisiae.*

**Materials and methods:**

*S. cerevisiae* strains FF18733 (wild-type), FF1481 (*rad1* mutant) and D7 (commonly used to detect reciprocal and nonreciprocal mitotic recombinations) were exposed to a 905 MHz electromagnetic field that closely matched the Global System for Mobile Communication (GSM) pulse modulation signals for mobile phones at a specific absorption rate (SAR) of 0.12 W/kg.

**Results:**

Following 15-, 30- and 60-minutes exposure to RF/MW radiation, strain FF18733 did not show statistically significant changes in colony growth compared to the control sample. The irradiated strains FF1481 and D7 demonstrated statistically significant reduction of colony growth compared to non-irradiated strains after all exposure times. Furthermore, strain FF1481 was more sensitive to RF/MW radiation than strain D7.

**Conclusions:**

The findings indicate that pulsed RF/MW radiation at a low SAR level can affect the rate of colony growth of different *S. cerevisiae* strains.

## Introduction

Microwave radiation is a type of non-ionizing electromagnetic radiation widely used in industry, commerce, medicine and for private purposes, especially in mobile communication. In recent years, the use of mobile phones has accelerated, resulting in increasing exposure of the environment to weak radiofrequency microwave (RF/MW) radiation generated by these devices. Although the average exposure levels are low compared to exposure limits, public concern about the potential hazard on human health is growing.^1^ Numerous experimental studies evaluating the biological effects caused by RF/MW radiation are controversial and no unanimous conclusion has been reached.^2^–^4^

It is well-documented that yeasts are representative of eukaryotes, including human cells, in many aspects of fundamental cellular processes.^5^ Many experiments, with the yeast *Saccharomyces cerevisiae* as a model organism, can be performed under biologically and technically well-controlled conditions after exposure to microwave radiation.^6^

The objective of this study was to evaluate the potential effect of 905 MHz RF/MW radiation similar to that emitted by mobile phones on colony growth of *S. cerevisiae* strains FF18733, FF1481 and D7.

## Materials and methods

### Yeast strains

This experiment was carried out using three *S. cerevisiae* strains. The FF18733 strain (*MATa leu2–3,112 trp1–289 ura3–52 his7–2 lys1–1*) is a wild-type, whereas the derived FF1481 strain (*MATa leu2–3,112 trp1–289 ura3–52 his7–2 lys1–1 rad1::LEU2*) is deficient in nucleotide excision repair.^7^ The D7 strain (*MATa/αade2–40/ade2–119 trp5–12/trp5–27 ilv1–92/ilv1–92*) is relatively genetically unstable. Therefore, changes in mitotic crossing-over, mitotic gene conversion and reverse mutations may occur spontaneously.^8^

### Experimental procedures

A preculture of three strains of *S. cerevisiae* was suspended in yeast extract liquid (YEL) and grown for 48 h at 28°C. Precultured cells (2 x 10^6^ cells/ml) were then suspended in YEL and grown for 18 h at 28°C. Half of each culture (2 x 10^8^ cells/ml), prepared in triplicate, were exposed to 905 MHz microwave radiation for 15, 30 and 60 minutes, whereas the other half served as a control. After radiation treatment, yeast cells were inoculated on solid complete growth medium and grown overnight at 28ºC. Thereafter, the number of colonies of the three strains (irradiated and non-irradiated) was counted under a magnifier.

### Exposure conditions

An electromagnetic field was generated using a Gigahertz Transversal Electromagnetic Mode Cell (GTEM-cell) model 5402 (ETS^TM^ Lindgren, USA) equipped with a signal generator (Antrisu MS27211B, Japan), signal amplifier (RF 3146 Power Amp Module, RF Micro Devices, Greensboro, USA) and a signal modulator (RF 2722 Polaris Chip, RF Micro Devices, Greensboro, USA). The signal amplifier was used to amplify the RF/MW signal induced by the signal generator, whereas the signal modulator was used to modulate a continuous wave to pulse signal used in the Global System for Mobile Communication (GSM) mobile phones. Yeast suspensions were exposed to 905 MHz RF/ MW with the GSM basic signal modulation for 15, 30 and 60 minutes. Inside the GTEM-cell, the electromagnetic field strength was 10 V/m, and the temperature was 28°C. The average specific absorption rate (SAR) for a single cell was 0.12 W/kg. SAR was calculated by averaging the individual parameters of the cell components in accordance with their volume fraction in live cells.^9^

### Statistical analysis

Statistical analyses were carried out with descriptive statistics. Significant differences in colony growth were determined using the Student’s t-test. Values of *P* lower than 0.05 were considered statistically significant.

## Results

**FIGURE 1:**
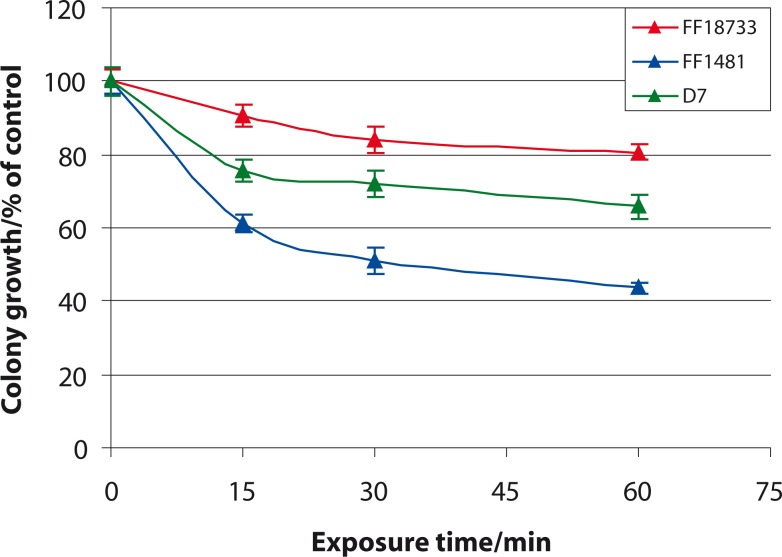
Colony growth of yeast *S. cerevisiae* strains FF18733, FF1481 and D7 after 15-, 30- and 60-minutes exposure to 905 MHz microwave radiation. Values represent means and standard deviations.

Figure 1 shows the colony growth of three *S. cerevisiae* strains after 15-, 30- and 60-minutes exposure to 905 MHz RF/MW radiation similar to that emitted by mobile phones at SAR of 0.12 W/kg. The number of non-irradiated colonies of each strain was taken as 100% and the percent of irradiated colonies after different exposure times was calculated with respect to this control sample. Following a 15-, 30- and 60-minutes exposure to RF/MW radiation, the wild-type strain FF18733 did not show statistically significant changes in colony growth compared to the control sample. Irradiated strains FF1481 and D7 demonstrated statistically significant reduction of colony growth compared to nonirradiated strains after all exposure times. The data indicate that RF/MW radiation decreased colony growth of strains FF18733, FF1481 and D7 resulting in a 19.30±2.06%, 56.37±1.49% and 34.29±3.21% growth reduction after 60-minutes exposure, respectively.

## Discussion

Users of mobile phones are exposed to weak microwave radiation. In this context, the possible effects of RF/MW radiation on genetic material are very important. Many studies on mammalian cells failed to find microwave-induced DNA damage and cell proliferation^10^–^12^; in contrast with exposure to ionizing radiation where the DNA damage is well known.^13^ Other studies have reported that modulated RF/MW radiation is capable of causing DNA lesions and inhibition of cell proliferation.^14^,^15^

In this study, we estimated the effect of mobile phones radiofrequency of 905 MHz on the yeast *S. cerevisiae* strains FF18733, FF1481 and D7. Strains FF1481 and D7 demonstrated a statistically significant difference in colony growth after 15-, 30- and 60-minutes exposure to pulsed RF/MW radiation at SAR 0.12 W/kg. Therefore, these strains showed increased sensitivity to RF/MW radiation and reduction of colony growth was time-dependent. An earlier experiment with the yeast *S. cerevisiae* demonstrated either an increased (up to 15%) or decreased (up to 38%) cell growth rate by certain frequencies of microwave radiation within a 41.6–41.8 GHz band.^16^–^18^

It is known that microwave radiation may occur directly by DNA lesion and/or indirectly by damage to DNA repair mechanisms. Strain FF1481 of *S. cerevisiae* is deficient in nucleotide excision repair due to an insertion of the functional *LEU2* gene at the *RAD1* locus and *rad1* becomes non-functional. Rad1, in complex with Rad10, exhibits single-stranded DNA endonuclease activity and cleaves 3’-ended single-stranded DNA at its junction with the duplex DNA.^19^ Since we observed a significant decrease of *rad1* mutant cell proliferation, it seems that pulsed RF/MW radiation at a low SAR level during short exposure times could induce DNA damage in *S. cerevisiae* cells.

Mitotic recombination is necessary during mitosis for the repair of DNA single- and double-strand breaks, and mutagenic lesions generated by exposure to chemicals and radiation.^20^ Strain D7 of *S. cerevisiae* is commonly used to detect reciprocal (crossing-over) and nonreciprocal (gene conversion) mitotic recombinations and reverse mutations. Besides evaluation of the RF/MW effect on cell growth rate of strain D7, we estimated the induction of mitotic gene conversion and reverse mutations in strain D7 after 15-, 30- and 60-minutes exposure to 905 MHz RF/MW at SAR 0.12 W/kg. The frequency of gene conversion at the *trp* locus and reverse mutation at the *ilv* locus showed only a slight tendency to increase compared to the control sample (data not shown). Preliminary results indicate that modulated RF/MW radiation with a low SAR value did not affect either the rate of gene conversion nor reverse mutations in strain D7. Gos *et al.*^21^ reported that mobile phone fields at 900 MHz with SAR of 0.13 and 1.3 W/kg did not exhibit any effect on mutations or recombinations in *S. cerevisiae* cells either in the absence or presence of genotoxic stress.

In conclusion, our study showed that three *S. cerevisiae* strains exhibit different patterns of colony growth after 15-, 30- and 60-minutes exposure to a mobile phones radiofrequency of 905 MHz at SAR 0.12 W/kg. Strains FF1481 (DNA repair mutant) and D7 (relatively genetically unstable) demonstrate an increased sensitivity to RF/MW radiation in comparison to strain FF18733 (wild-type). The data indicate that pulsed RF/MW radiation at a low SAR level could induce DNA damage in *S. cerevisiae* cells. This points to the need for further studies of DNA repair mechanisms in yeast cells.
